# Post-translational modifications of FDA-approved plasma biomarkers in glioblastoma samples

**DOI:** 10.1371/journal.pone.0177427

**Published:** 2017-05-11

**Authors:** Natalia A. Petushkova, Victor G. Zgoda, Mikhail A. Pyatnitskiy, Olesya V. Larina, Nadezhda B. Teryaeva, Alexander A. Potapov, Andrey V. Lisitsa

**Affiliations:** 1 V.-N. Orekhovich Research Institute of Biomedical Chemistry, Moscow, Russia; 2 N.-N. Burdenko Research Institute of Neurosurgery, Moscow, Russia; Swiss Institute of Bioinformatics, SWITZERLAND

## Abstract

Liquid chromatography-tandem mass spectrometry was used to analyze plasma proteins of volunteers (control) and patients with glioblastoma multiform (GBM). A database search was pre-set with a variable post-translational modification (PTM): phosphorylation, acetylation or ubiquitination. There were no significant differences between the control and the GBM groups regarding the number of protein identifications, sequence coverage or number of PTMs. However, in GBM plasma, we unambiguously observed a decreased fraction in post-translationally modified peptides identified with high quality. The disease-specific PTM patterns were extracted and mapped to the set of FDA-approved plasma protein markers. Decreases of 46% and 24% in the number of acetylated and ubiquitinated peptides, respectively, were observed in the GBM samples. Significance of capturing disease-associated patterns of protein modifications was envisaged.

## Introduction

Post-translational modifications (PTMs) of proteins affect pathways linked to cell signaling/transduction, trafficking, storing, expression, binding and/or affinity and cause serious health consequences including cancer. PTMs in cancer include phosphorylation, acetylation, methylation, glycosylation, and ubiquitination [1]. Most PTMs alter the molecular mass of a protein, therefore, mass spectrometry (MS) is the ideal analytical tool for PTM profiling of proteomes [2]. However, PTMs can rigorously compromise protein identification results if proper accounting of combinatorial variants is not performed. Although database search engines support options to identify peptides with specific modifications, search algorithms cannot comprehensively identify PTMs in a single pass because of high false discovery rates (FDRs) [3, 4].

The validation of the protein match to the mass-spectra usually relies on number of identified peptides, semi-probabilistic scores and sequence coverage. In liquid chromatography-tandem MS (LC-MS/MS) experiments the sequence coverage roughly correlates with protein abundance. A highly sensitive shotgun approach to peptide detection (below fmol levels) is characterized by low level of protein sequence coverage, which is a limitation of the bottom-up approach in the characterization of PTMs of proteins [5].

There is no standardized procedure for PTM analysis; further advances are required in protein isolation, MS sensitivity and MS/MS data processing. The use of multiple proteases could increase protein sequence coverage, thus increasing the probability to find PTMs [6]. Affinity-based enrichment, for example, immobilized metal ion affinity chromatography or immunoaffinity and MS, make it possible to perform PTM characterization. However, even with specific enrichment, only a few types of PTMs can be identified presumably by phosphorylation [7]. The simplest strategy for identifying PTMs from peptide MS data is running a “variable modification search”, in which a particular PTM (e.g., phosphorylation) is may occur on any instance of selected amino acid residues (e.g., serine, threonine, or tyrosine) in all of the theoretical peptides from the entire search database. Otherwise, it is possible to run MS/MS search, which enables to identify a range of modifications [8]. For example, Chick et al. [9] reported that high-resolution MS/MS spectra achieved by instruments, such as the Orbitrap, often supply sufficient information to match modified peptides in so called “open searches”, that is, when the precursor ion tolerance vastly increases, while the fragment ion tolerance is narrow (0.01 Da). The authors confidently matched the proteome-wide dataset on HEK293 cells (9,513 proteins and 396,736 peptides) to an additional 184,000 modified peptides by a Sequest database search with a ± 500 Da precursor ion tolerance. In our work, we searched for PTMs using commercial engine Mascot (Matrix Science) with which the separate searches were performed with the variable mode either switched off or specified as acetylation, phosphorylation or ubiquitination.

For this study, the glioblastoma multiforme (GBM) plasma samples were selected because this tumor possesses high heterogeneity, which complicates therapeutic intervention [10]. The importance of phosphorylation processes as regulators of pathways in GBM stem cells was found [11]. GBM treatment is connected with histone deacetylase inhibitors, which have shown promise for improving patient outcomes [12]. Removing acetyl groups from the lysines of histone and nonhistone proteins alters transcription and promotes alternative post-translational lysine modifications such as methylation, ubiquitination, and phosphorylation [13].

The aim of this study was the proteomic profiling of post-translationally modified peptides of plasma proteins in GBM. A multiple MS/MS search was used to produce the PTM-patterns for FDA-approved plasma proteins [14] in GBM patients. We found that unravelling modified peptides exhibit disease-specific patterns of PTMs, which putatively could indicate disease risk, and the efficacy of drug therapy.

## Materials and methods

### Materials

All chemicals used in this study were HPLC-grade. Sequencing grade modified trypsin (V5111) was obtained from Promega (Madison, WI, USA); 2,2-bicinchoninic acid was from Pierce (Rockford, IL, USA). Other reagents and solvents were from Sigma-Aldrich and Acros Organics.

### Clinical samples

Patient recruitment and sample collection protocols were approved by the local ethics rules of the Burdenko Research Institute of Neurosurgery (Moscow, Russia). Informed consent was obtained from all patients. In total, 24 blood samples were gathered; twelve blood samples were collected from patients (seven men and five women, age between 25 and 66 years) prior to any treatment. All patients enrolling in the study were hospitalized with a first diagnosis of a primary high-grade glioma (grade IV or at least grade III). Afterwards tumors had been assigned histologically to glioblastoma (GBM, WHO grade IV). In addition, 12 blood samples (denoted as Control) were collected from age-matched healthy volunteers (age between 17 and 62 years, five men and seven women). Each blood sample was screened for viral and bacterial infection markers of blood-transmittable diseases (HIV, hepatitis B, hepatitis C and syphilis). Venous blood samples were obtained after an overnight fast into EDTA tubes and centrifuged in a J2-21 centrifuge at 1,500g x 10 min at room temperature. The plasma was stored at −80°C in cryotubes until further processing.

### Depletion of high abundant proteins from human plasma

The depletion was performed using Albumin & IgG Depletion Kit according to the manufacturer’s instructions (Sigma-Aldrich). Total protein concentration of depleted plasma samples was determined with 2,2-bicinchoninic acid [15], using bovine serum albumin (BSA) as a standard.

### In-solution tryptic digestion

The depleted samples (175 μg of protein) for each GBM (n = 12) or control (n = 12) plasma were in-solution digested in accordance with a standard protocol [16]. In brief, protein denaturation and disulfide bonds reduction was performed with a solution containing sodium deoxycholate, TCEP (Tris (2-carboxyethyl)phosphine hydrochloride), and 1,4-dithiothreitol, and alkylated with vinylpyridine. A 9.9 μL aliquot of 200 ng/ μL trypsin solution was added to the sample and incubated at 44°C for 2 h, after which another 11.4 μL of trypsin was added, and the solution incubated at 37°C for an additional 2 h, then the enzymatic digestion was stopped by the addition of 9.6 μL of formic acid and then centrifuged (15 min).

### LC-MS/MS analysis

Separation and identification of the peptides were performed on a Ultimate 3000 nano-flow HPLC (Dionex, USA) connected to Orbitrap Exactive (Thermo Scientific) mass spectrometer equipped with a Nanospray Flex NG ion source (Thermo Scientific). 1 μL of Peptide separation was carried out on a RP-HPLC column Zorbax 300SB-C18 (C18 particle size of 3.5 μm, inner diameter of 75 μm and length of 150 mm) using a linear 90-min gradient from 95% solvent A (water, 0.1% formic acid) and 5% solvent B (water, 0.1% formic acid, and 80% acetonitrile) to 60% solvent B over 95 min at a flow rate of 0.3 μL/min.

Mass spectra were measured in the positive ion mode. Data was acquired in the Orbitrap Exactive analyzer with resolution of 70,000 (at m/z 400) for MS and 15,000 (m/z 400) for MS/MS scans. Survey MS scan was followed by MS/MS spectra acquisition for the ten most abundant precursors. For peptide fragmentation higher energy collisional dissociation (HCD) was used, the signal threshold was set to 17,500 for an isolation window of 1 m/z and the first mass of HCD spectra was set to 100 m/z. The collision energy was set to 35%. Fragmented precursors were dynamically excluded from targeting for 10 s. Singly charged ions and ion with not defined charge state were excluded from triggering MS/MS scans. Three independent LC-MS/MS runs were performed for each sample.

We acknowledge the IBMC “Human Proteome” Core Facility for assistance with the generation of mass-spectrometry data.

### Data processing

A total of 36 LC-MS/MS runs were carried out for blood-depleted plasma from GBM patients as well as control. Raw files were merged into two mgf-files by Progenesis LC-MS software (Nonlinear Dynamics Ltd.). Each of the mgf files containing feature list for protein identification searched by Mascot software (www.matrixscience.com). Searched results were re-imported into Progenesis LC-MS to assign chromatographic featured with protein identifications.

Protein identification in Mascot software was performed with decoy [17] against SwissProt (SP, 2012_11 version, .fasta format) for Homo sapiens. Trypsin was specified as the proteolytic enzyme; up to 1 or 2 missing cleavages were allowed. Pyridylethylation (C) was used as static modification, while oxidation of methionine was set as variable. To examine the phosphorylation, acetylation or ubiquitination status of the proteins/peptides Mascot search was additionally performed with one of the following modifications setting as variables: phosphorylation of serine, threonine and tyrosine (Phospho S, T, Y); acetylation of lysine and N-terminal of proteins (Acetyl K, protein N-term); or ubiquitination of lysine (GlyGly K). Charge states of +2, +3, and +4 were taking into account for parent ions. Mass tolerance was set to ± 15 ppm for parent ion masses and ± 0.01 Da for fragment ion masses; false discovery rate (FDR) ≤ 1%. The peptides reported by Mascot with significance index (SI) > 13 were selected for separate analysis.

Statistical analysis was performed using R software [www.r-project.org]. The distributions of proteins sequence coverage were analyzed via Anderson-Darling and Shapiro-Wilk normality tests using functions “ad.test” and “shapiro.test” from packages “nortest” and “stats”, respectively. Comparisons between control and GBM samples across various types of PTMs were evaluated with two-sided Wilcoxon test (wilcox.test function). Violin plots were generated using 'beanplot' package [18]. Comparison of fractions of post-translationally modified proteins and peptides was performed using “prop.test” function.

Reproducibility (repeatability) for the three replicate LC-MS/MS runs per sample was calculated as accordance, i.e., the chance that two identical test materials analyzed by the same laboratory under standard repeatability conditions will both be given the same result [18a]. This metric was used due to its capability to deal with the qualitative (Bernoulli) data. Calculations were performed according the formula 1 from the Langton et al [19], using in-house developed PHP-script [http://php.net/].

$$\text{accordance}=\frac{\text{k}\left(\text{k}-1\right)+\left(\text{n}-\text{k}\right)\left(\text{n}-\text{k}-1\right)}{\text{n}\left(\text{n}-1\right)},$$ (1)

where k—number of runs per sample where particular protein was identified; n—total number of runs per sample.

## Results and discussion

Due to widespread recognition of the importance of PTMs [20] nearly all MS/MS search algorithms support a PTM-sensitive mode. The standard strategy for identifying PTMs from peptide MS/MS data is a “variable modification search”, in which a particular PTM (e.g., phosphorylation) is allowed to occur on selected amino acid residues (e.g., serine, threonine, or tyrosine) in all of the theoretical peptides from the entire search database [3].

### Analysis of GBM plasma proteome upon PTM-sensitive search

The examination of the plasma proteome was performed by Progenesis LC-MS software (Nonlinear Dynamics Ltd.), with which two peak lists were generated and exported for MS/MS search. We used Mascot engine, to perform a database search for possible modifications enabled using a target_decoy approach for extracting PTM peptides with FDR ≤ 1%. First, the search was carried out, restricting for any PTM. Second, we separately searched the same raw data for the cases in which variable modifications were allowed to be acceptable but only for one modification at a time. That modification was phosphorylation of every serine, lysine, and tyrosine, either acetylation of every lysine and protein N-term or ubiquitination of every lysine. Therefore, we obtained the variable modifications as separate sets of search results. Thirdly, we checked for PTM patterns specific to the control versus GBM plasma samples.

The lists of identified proteins for the control and GBM plasma samples (including number of peptides per protein, score, sequence coverage and normalized protein abundances) are given in S1–S8 Tables.

LC-MS/MS analysis revealed 3,227 peptides, which represented 446 proteins in the control plasma. This basic list of identified proteins was denoted as noPTMs, because the MS/MS search was conducted by prohibiting any modifications. Among the reported proteins, ~32% were represented by a single peptide only. For GBM plasma, 4,642 peptides and 597 proteins were determined (Columns 1 and 5, S9 Table); 28% of these were represented by single peptide.

Shotgun proteomics is not a high reproducibility method due to involving a complex mixture of proteins (e.g., purified from an organelle or associated with a particular disease state), cutting the proteins into peptides by sequence-specific proteolysis, separation of resulting peptides by LC and then analyzing the mixture of peptides using MS, for example tandem MS [21]. Therefore, to establish the repeatability of the protein identifications each sample was measured in triplicate. Repeatability for the three replicate LC-MS/MS runs per sample was calculated as accordance: the chance (percentage) that replicates analyzed under repeatability conditions would produce the same list of identified proteins/peptides [22]. The accordance for protein identifications was the average (mean) of these probabilities for each sample, and was 86±27% for control and 84±29% for GBM plasma.

Expanding the database search with possible protein/peptide phosphorylation (“Phospho”), acetylation (“Acetyl”) or ubiquitination (“GlyGly”) expectedly led to an increase in the number of identified proteins in the control plasma. A total of 3,649, 5,871 and 5002 peptides, were assigned to 519, 878 and 739 proteins, respectively, were observed dependently on the type of modification. The number of identified proteins in GBM plasma samples was significantly lower: 402 phosphorylated, 466 acetylated and 597 ubiquitinated species (S9 Table). Therefore, modification-sensitive MS/MS searching resulted in a decreased number of identified peptides and proteins in the depleted plasma of GBM patients in comparison to the noPTMs search.

Variable modifications did not affect the fraction of proteins identified by one peptide. According to the data in S9 Table, the portion of proteins revealed by a single peptide was on average 29.5±2.1% for the control (n = 4, where n was noPTMs + three PTM datasets) and 27.8±1.0% (n = 4) for the GBM, which was not a significant difference (p˃0.05). There was also no difference between the control and GBM samples in terms of the average numbers of peptides (4,437 ± 1,219 *vs*. 4,111 ± 627) or proteins identified by two peptides (8±1% *vs*. 10± 2%). The fractions of non-quantifiable proteins, for which Progenesis LC-MS generated no quantitative data, for both the control and GBM samples were approximately the same (40 ± 8% *vs* 35 ± 5%).

One or two protein-specific peptides are typically enough to confirm the presence of a protein within a sample. However, to characterize the protein by PTMs, higher sequence coverage is required [23]. For further analysis, we selected only identifications, that were made by multiple peptides (≥ 3). For such identifications, an average sequence coverage was ~36% for proteins from the control and GBM plasma (S9 Table): 34%–37% as the mean and 31–35 as the median. These observations closely match those of the literature data [24, 25].

**Fig 1:**
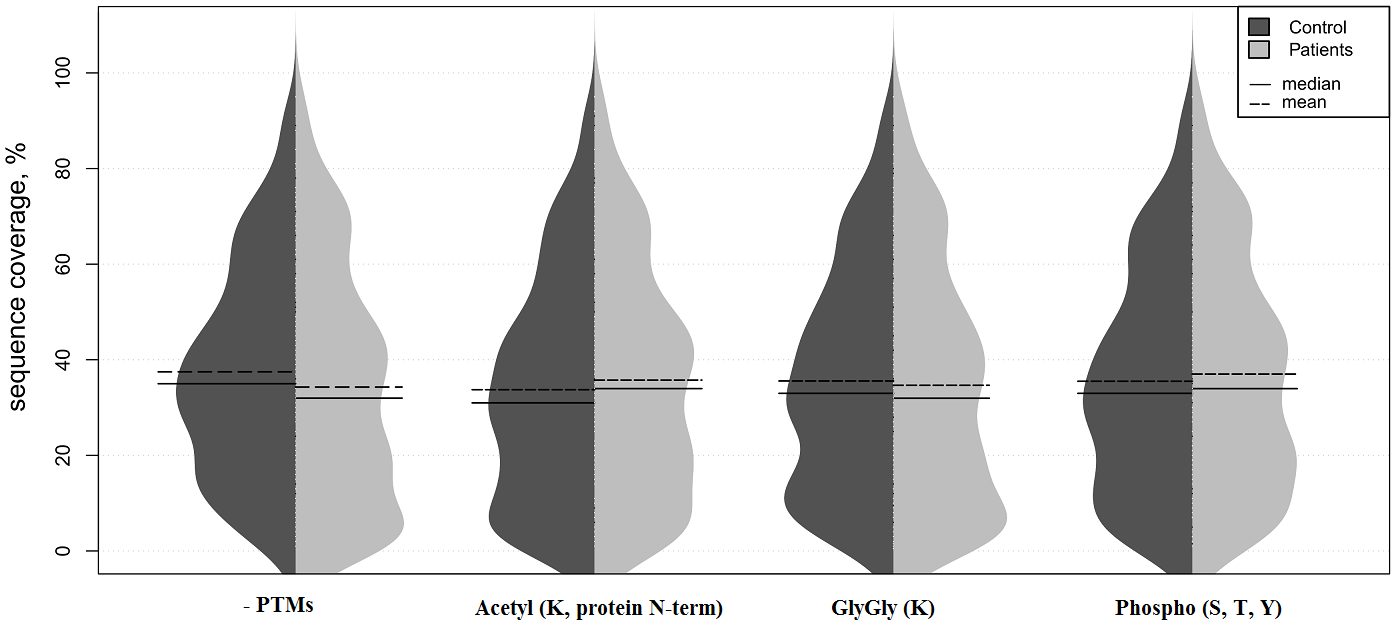
Sequence coverage for various protein PTMs. Identification by Mascot of ≥ 3 peptides per protein. The adjusted inverse normalized values are displayed as a violin plot, comparing distributions between control and GBM samples. Horizontal bars indicate the mean (dashed line) and median (solid line) values for each group.

We used a violin plot [26] to illustrate that the sequence coverage distributions showed no difference in all four types of modifications (Fig 1). So, on average there were no marked differences in the MS/MS search results due to PTM settings or samples’origin (Table 1, Fig 1 and S9 Table). It is possible to conclude that PTM and noPTM datasets were both equal in terms of the quality of MS/MS search results.

**Table 1:** Number of identified proteins/peptides due to PTM-sensitive MS/MS search for control and GBM plasma samples.

Parameter	Portion of proteins identified by ≥ 3 high quality peptides*		Portion of quantified peptides	
	Control	GBM	Control	GBM
noPTMs	28%	23% (– 5%)	69%	53% (– 4%)
Phospho	27%	32% (+ 5%)	61%	69% (+ 8%)
Acetyl	16%	28% (+ 12%)	36%	63% (+ 7%)
GlyGly	19%	23% (+ 4%)	44%	53% (+ 9%)

noPTMs—search with no PTMs allowance; Phospho—search with possible protein phosphorylation; Acetyl—search with possible acetylation; GlyGly—possible ubiquitination.

* Peptides automatically selected for identification and quantification by Progenesis LC software.

We observed two simultaneous events for identifications by multiple peptides due to the search parameters (i.e., with noPTMs or PT set) in control and GBM samples (Table 1). The fraction of proteins represented by ≥3 peptides was increased in the GBM samples when the search was performed with possible phosphorylation (Phospho), p = 0.004; and did not change when the search was performed with possible acetylation (Acetyl) or ubiquitination (GlyGly) (p-0.088 and 1.000, respectively). Conversely, when phospho-target searching was performed in the controls and compared to the noPTMs search, the difference was insignificant (p = 0.621) and significant when the search was performed with possible acetylation or ubiquitination (p<0.001 in both cases). We cannot say whether or not these observations are essential for interpretation of MS/MS search reports. To capture PTMs, it is usually necessary to widen the window for the parent ion to make it incorporable with the peaks that are shifted from the original mass due to possible modifications. Such a shift can sufficiently influence the FDR due to the growth of combinatorial complexity. In our experiments, we controlled for FDR in the range of 0.74–0.82% for all searches (S9 Table).

We compared the number of highly confident identifications normalized to the total number of proteins in particular PTM/noPTMs datasets. From Table 1 it is possible to expect an increase in the quality of identifications in GBM due to an increase in the percentage of the proteins identified by multiple peptides at PTM-sensitive search up to 28±5% from 23% for noPTMs. Although the fraction of no PTMs proteins was higher in the controls, the fraction of proteins identified by ≥3 high-quality peptides for phosphorylation was higher in GBM as compared to the controls. However, for these cases the difference was insignificant (p = 0.083 and 0.098, respectively). The difference in the proportion of identified proteins in the GlyGly search was nearly significant (p = 0.0054). In the case of Acetyl, 16% of the proteins were identified by ≥3 peptides in the control and 28% in the GBM samples; it is the only case when the difference was statistically relevant (p˂0.001, Table 1).

### Modified peptides in GBM samples

**Fig 2:**
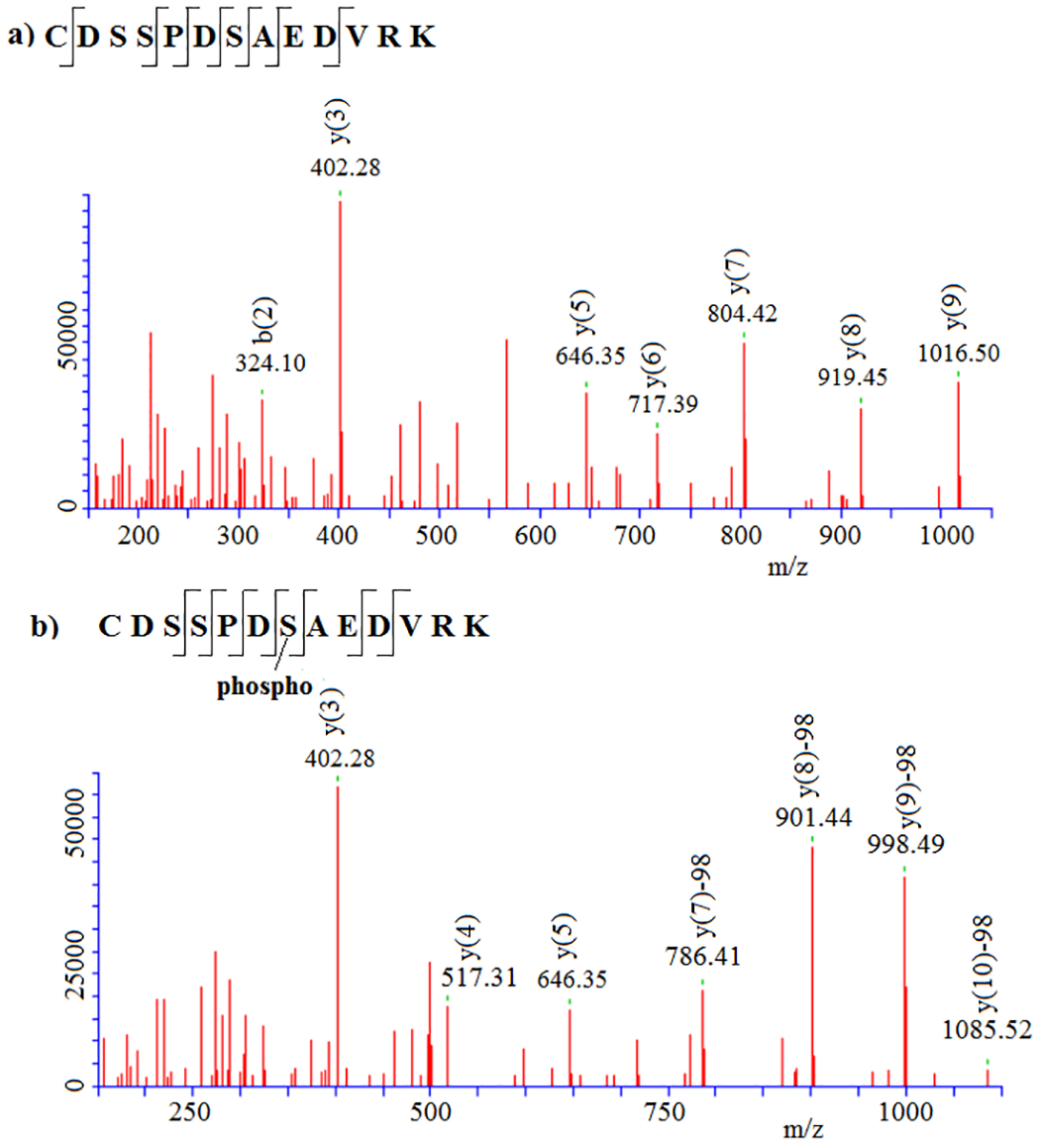
MS/MS spectra of non-modified (A) and phosphomodified (B) peptide DSSPDSAEDVR (2+) of alpha-2-HS-glycoprotein (FETUA_HUMAN) GBM plasma. Monoisotopic mass of neutral peptide Mr (calc) 1,592.62. Variable modification S7–Phospho (ST) with neutral loss 97.98. [*y*(10)] in a non-modified peptide was not detected.

Analysis of the Mascot and Progenesis LC-MS reports revealed differences for PTM-specific MS/MS data linked to the presence or absence of modified peptides in GBM samples, as compared to the controls. The example of alpha-2-HS-glycoprotein 2 (FETUA) is illustrated in Table 2. Seventeen peptides (≥7 aa) were detected in the control plasma. Of these, 12 peptides were observed with no possible protein/peptide modifications. Considering possible phosphorylation (Phospho S, T, Y), we observed three additional peptides (nos. 8, 10 and 16) for FETUA. The appended data revealed by PTM-targeted search increased the sequence coverage by ~4%. The MS/MS spectra of the representative phosphopeptide no. 10 and corresponding unmodified peptide no. 9 (CDSSPDSAEDVR) with respective reporter ions are shown in Fig 2. The detection of the fragment phospho ion *y(7)* in the peptide ^132^Ser-Arg^144^ enabled unambiguous identification of the phosphorylation site at the Ser^138^ residue for alpha-2-HS-glycoprotein.

**Table 2:** Enrichment of plasma Alpha-2-HS-glycoprotein (FETUA_HUMAN, 367 aa) sequence coverage with peptides assembled from MS/MS search for phosphorylated peptides.

## no	Start-End	Peptide	Mr (expt)	noPTMs^1^		Phospho (S,T,Y)^2^	
				Control	GBM	Control	GBM
**1**	29–57	R.QPNCDDPETEEAALVAIDYINQNLPWGYK.H	3410.58	+	+	+	+
**2**	58–67	K.HTLNQIDEVK.V	1195.62	+	+	+	+
**3**	58–99	K.HTLNQIDEVKVWPQQPSGELFEIEIDTLETTCHVLDPTPVAR.C	4902.45	+	*nd*	+	*nd*
**4**	68–99	K.VWPQQPSGELFEIEIDTLETTCHVLDPTPVAR.C	3724.85	+	+	+	+
**5**	104–120	R.QLKEHAVEGDCDFQLLK.L	2077.03	+	+	+	+
**6**	107–120	K.EHAVEGDCDFQLLK.L	1707.79	+	+	+	+
**7**	132–143	K.CDSSPDSAEDVR.K	1384.56	+	+	*nd*	*nd*
**8**	132–143	K.CDSSPDSAEDVR.K + Phospho (ST)	1464.52	*nd*	*nd*	+	+
**9**	132–144	K.CDSSPDSAEDVRK.V	1512.65	+	+	+	+
**10**	132–144	K.CDSSPDSAEDVRK.V + Phospho (ST)	1592.62	*nd*	*nd*	+	+
**11**	144–159	R.KVCQDCPLLAPLNDTR.V + Phospho (ST)	2075.00	*nd*	*nd*	*nd*	+
**12**	188–211	R.AQLVPLPPSTYVEFTVSGTDCVAK.E	2626.34	*nd*	+	*nd*	+
**13**	219–225	K.CNLLAEK.Q	894.46	+	+	+	+
**14**	226–231	K.QYGFCK.A	849.46	+	+	+	+
**15**	318–337	R.HTFMGVVSLGSPSGEVSHPR.K	2080.02	+	+	+	+
**16**	318–337	R.HTFMGVVSLGSPSGEVSHPR.K + Phospho (ST)	2159.98	*nd*	–	+	+
**17**	341–361	R.TVVQPSVGAAAGPVVPPCPGR.I	2063.01	+	+	+	+
**Sequence coverage**				42%	48%	46%	52%

Notes:

^1^ –Mascot searching without taking into account possible PTMs;

^2^ –Mascot searching with taking into account possible protein phosphorylation (Phospho S,T,Y)

*nd*—not detectable

Analyzing FETUA peptides, we noted that 10 unmodified peptides with nos. 1, 2, 4–6, 9, 13–15 and 17 were revealed in the control and GBM samples. As for phosphopeptides nos. 8, 10 and 17 were also found in both types of samples. Table 2 shows that there was a single modified peptide (no. 11) absent in the control plasma but present in GBM samples. This means that this modified peptide could be used as a component of the PTM pattern that separates groups of samples.

We also observed the reverse situation when a modified peptide was presented in the control and absent in the GBM. For example, a PTM-sensitive search did not reveal acetylated and phosphorylated peptides of phosphatidylcholine-sterol acyltransferase (LCAT_HUMAN) in GBM samples, but such modifications were observed in the controls.

### Modified peptides for FDA-approved plasma biomarkers

By implementing LC-MS/MS with control and GBM plasma, we identified 7,221 peptides, which represented ~1,300 proteins and ˃1000 peptides were modified. Among these proteins, we detected 37 FDA-approved biomarkers [14]. There are several preliminary communications that some of these biomarkers (e.g. serum albumin, serum alpha 1-acid glycoprotein, and C-reactive protein) provide prognostic information in patients with GBM [27–29]. Most identified FDA-approved protein markers were quantified in all control and GBM plasma samples. Only transferrin receptor protein 1 was absent in the control list of proteins identified using the noPTMs search mode.

One hundred unique PTM peptides proteotypic for FDA plasma markers were identified in the control plasma samples. These included 33 phosphorylated, 38 acetylated and 29 ubiquitinated peptides (Table 3). We observed a decrease in the number of modified peptides: up to 75 in the GBM compared to 100 in the control samples, however, this difference was not significant (p = 0.061). Further analysis revealed changes in the number of acetylated and ubiquitinated peptides of FDA biomarkers in the GBM and control samples. The number of ubiquitinated peptides decreased by 24% in the GBM samples, whereas there was a 46% reduction in the number of acetylated peptides.

**Table 3:** List of modified peptides of FDA-approved proteins identified in control and GBM plasma via PTM-sensitive MS/MS search (Mascot).

##	Accession	Modified peptide	Control plasma			GBM plasma		
			Phospho (S,T,Y)^1^	Acetyl (K, protein N-term)^2^	GlyGly (K)^3^^,^*	Phospho (S,T,Y)^1^	Acetyl (K, protein N-term)^2^	GlyGly (K)^3^^,^*
**1**	ALBU_HUMAN (Serum albumin)	+AFKAWAVAR *[N-term] Acetyl (N-term)|*[3] *Acetyl (K)*		+			*nd*	
		VFDEFKPLVEEPQNLIKQNCELFEQLGEYK [6] *Acetyl (K)*		+			*nd*	
		SEVAHRFK [8] *Acetyl (K)*		*nd*			+	
		HKPKATK [2] *GlyGly (K)|*[4] *GlyGly (K)*			+			*nd*
**2**	APOA_HUMAN (Apolipoprotein(a)	IPLYYPNAGLTRNYCR [5] *Phospho (Y)|*[11] *Phospho (ST)|*[14] *Phospho (Y)*	*nd*			+		
		IPLYYPNAGLTR [5] *Phospho (Y)*	*nd*			+		
		SPVVQDCYHGDGR [1] *Phospho (ST)*	+			*nd*		
**3**	A1AT_HUMAN (Alpha-1-antitrypsin)	VFSNGADLSGVTEEAPLK [3] *Phospho (ST)*	*nd*			+		
		FNKPFVFLMIEQNTKSPLFMGK [22] *Acetyl (K)*		+			*nd*	
		FNKPFVFLMIEQNTK [15] *GlyGly (K)*			+			+
		LSITGTYDLKSVLGQLGITKVFSNGADLSGVTEEAPLK [20] *GlyGly (K)*			+			*nd*
		QINDYVEKGTQGKIVDLVK [13]*GlyGly(K)*[19]*GlyGly (K)*			*nd*			+
**4**	A2MG_HUMAN (Alpha-2-macroglobulin)	KLSFYYLIMAK [11] *Acetyl (K)*		+			*nd*	
		KDTVIKPLLVEPEGLEK [17] *Acetyl (K)*		*nd*			+	
		VTGEGCVYLQTSLK [14] *GlyGly (K)*			*nd*			+
**5**	A2AP_HUMAN (Alpha-2-antiplasmin)	LVPPMEEDYPQFGSPK [9] *Phospho (Y)*	+			+		
		MYLQKGFPIK [5] *GlyGly (K)*			+			*nd*
**6**	ANT3_HUMAN (Antithrombin-III)	YSNVIGTVTSGK [12] *Acetyl (K)*		+			*nd*	
		RVWELSK [7] *Acetyl (K)*		*nd*			+	
		YSNVIGTVTSGKRK [14] *GlyGly (K)*			+			+
**7**	APOA1_HUMAN (Apolipoprotein A-I)	+DLATVYVDVLK *[N-term] Acetyl (N-term)*		+			*nd*	
**8**	C1QC_HUMAN (Complement C1q subcomponent subunit C)	GPKGQK [3] *GlyGly (K)|*[6] *GlyGly (K)*			+			*nd*
		DGYDGLPGPKGEPGIPAIPGIR [10] *GlyGly (K)*			*nd*			+
**9**	APOB_HUMAN (Apolipoprotein B-100)	FSLDGKAALTELSLGSAYQAMILGVDSK [27] *Phospho (ST)*	+			*nd*		
		FSLDGKAALTELSLGSAYQAMILGVDSK [18] *Phospho (Y)*|[27] *Phospho (ST)*	*nd*			+		
		SVSLPSLDPASAK [6] *Phospho (ST)*	*nd*			+		
		GSWKWACPR [2] *Phospho (ST)*	+			+		
		LTISEQNIQRANLFNK [4] *Phospho (ST)*	+			+		
		QSMTLSSEVQIPDFDVDLGTILRVNDESTEGK [4] *Phospho (ST)*	+			*nd*		
		QSMTLSSEVQIPDFDVDLGTILRVNDESTEGK [29] *Phospho (ST)*	+			+		
		TTLTAFGFASADLIEIGLEGKGFEPTLEALFGK [4] *Phospho (ST*)|[10*] Phospho (ST*)|[26] *Phospho (ST)*	+			+		
		YGMVAQVTQTLKLEDTPK[10]*Phospho (ST*)[16]*Phospho(ST)*	+			+		
		YKLQDFSDQLSDYYEK [13] *Phospho (Y*)|[14] *Phospho (Y*)	*nd*			+		
		YKLQDFSDQLSDYYEK [7] *Phospho (ST*)|[14] *Phospho (Y)*	+			*nd*		
		YKLQDFSDQLSDYYEK [11] *Phospho (ST)|*[14]*Phospho (Y)*	+			*nd*		
		SSVKLQGTSK [9] *Phospho (ST)*	*nd*			+		
		+CVQSTKPSLMIQK *[N-term] Acetyl (N-term)|*[6] *Acetyl (K)*[13] *Acetyl (K)*		+			*nd*	
		ELLKDLSK [4] *Acetyl (K)*		+			*nd*	
		FLDSNIK [7] *Acetyl (K)*		*nd*			+	
		HVGSKLIVAMSSWLQK [16] *Acetyl (K)*		+			+	
		HVGSKLIVAMSSWLQK [5] Acetyl (K)		*nd*			+	
		+IPSVQINFK *[N-term] Acetyl (N-term)*		+			*nd*	
		+NKYGMVAQVTQTLK *[N-term]Acetyl (N-term)*[2]*Acetyl (K)*		+			*nd*	
		YLRTEHGSEMLFFGNAIEGK [20] *Acetyl (K)*		+			+	
		EFLKTTK [4] *GlyGly (K)*			+			*nd*
		FLDSNIK [7] *GlyGly (K)*			+			+
		FSHVEKLGNNPVSK [6] *GlyGly (K)*			+			*nd*
		FSHVEKLGNNPVSK [14] *GlyGly (K)*			+			*nd*
		NEIKTLLK [4] *GlyGly (K)|*[8] *GlyGly (K*)			+			*nd*
		TLQELK [6] *GlyGly (K)*			+			+
		YEGLQEWEGK [10] *GlyGly (K)*			+			+
		FVEGSHNSTVSLTTKNMEVSVATTTK [15] *GlyGly (K)* [26] *GlyGly (K)*			*nd*			+
		KMGLAFESTK [10] *GlyGly (K)*			*nd*			+
**10**	BTD_HUMAN (Biotinidase)	FGIFTCFDILFFDPAIR [5] *Phospho (ST)*	+			*nd*		
		FVVCIMSGARSK [7] *Phospho (ST*)|[11] *Phospho (ST)*	*nd*			+		
		LSCMAIRGDMFLVANLGTK [19] *GlyGly (K)*			*nd*			+
**11**	CERU_HUMAN (Ceruloplasmin)	TYCSEPEKVDK [11] *Acetyl (K)*		+			*nd*	
**12**	CHLE_HUMAN (Cholinesterase)	FSEWGNNAFFYYFEHR [12] *Phospho (Y)*	+			+		
		YLTLNTESTRIMTK [14] *Acetyl (K)*		+			+	
		FLFWFLLLCMLIGK [14] *GlyGly (K)*			+			*nd*
**13**	C1S_HUMAN (Complement C1s subcomponent)	EDTPNSVWEPAKAK [12] *Acetyl (K)*		*nd*			+	
**14**	C1R_HUMAN (Complement C1r subcomponent)	KEFMSQGNK [5] *Phospho (ST)*	+			+		
		+LRYTTEIIK *[N-term] Acetyl (N-term)*		+			*nd*	
		+NRMDVFSQNMFCAGHPSLK *[N-term] Acetyl (N-term)*		+			*nd*	
		IIGGQKAK [6] GlyGly (K)			+			*nd*
		KEFMSQGNK [9] *GlyGly (K)*			*nd*			+
		WVATGIVSWGIGCSRGYGFYTKVLNYVDWIK [22] *GlyGly (K)|*[31] *GlyGly (K)*			*nd*			+
**15**	CO3_HUMAN (Complement C3)	LPRGCGEQTMIYLAPTLAASR [9] *Phospho (ST*)|[20] *Phospho (ST*)	+			*nd*		
		LPRGCGEQTMIYLAPTLAASR [12]*Phospho (Y)|*[16] *Phospho (ST)*	+			*nd*		
		FDLKVTIKPAPETEK [4] *Acetyl (K) |*[8] *Acetyl (K)* [15] *Acetyl (K)*		+			*nd*	
		FLTTAKDK [8] *Acetyl (K)*		+			*nd*	
		FYHPEKEDGK [6] *Acetyl (K)*		+			+	
		FYHPEKEDGK [10] *Acetyl (K)*		+			+	
**16**	CO4A_HUMAN (Complement C4-A)	LPRGCGEQTMIYLAPTLAASR [9] *Phospho (ST*)|[20] *Phospho (ST)*	+			*nd*		
		LPRGCGEQTMIYLAPTLAASR [12] *Phospho (Y)|*[16] *Phospho (ST)*	+			*nd*		
		+DHAVDLIQK *[N-term] Acetyl (N-term)|*[9] *Acetyl (K)*		+			*nd*	
		YLDKTEQWSTLPPETK [4] *Acetyl (K)*		*nd*			+	
		YLDKTEQWSTLPPETK [16] *Acetyl (K)*		+			+	
**17**	CO5_HUMAN (Complement C5)	VSITSITVENVFVK [7] *Phospho (ST)*	+			*nd*		
		NFKNFEITIK [8] *Phospho (ST)*	*nd*			+		
		FWKDNLQHKDSSVPNTGTAR [3] *GlyGly (K)* [9] *GlyGly (K)*			+			*nd*
		NFKNFEITIK [3] *GlyGly (K)*			+			*nd*
		QLRLSMDIDVSYK [13] *GlyGly (K)*			+			*nd*
		VLGQVNK [7] *GlyGly (K)*			+			+
		YGMWTIKAK [7] *GlyGly (K)*			+			*nd*
**18**	CRP_HUMAN (C-reactive protein)	APLTKPLK [5] *Acetyl (K)*		+			*nd*	
**19**	FA9_HUMAN (Coagulation factor IX)	SCEPAVPFPCGR [1] *Phospho (ST)*	*nd*			+		
		ILNRPKR [6] *Acetyl (K)*		+			+	
		YVNWIKEKTK [6]*GlyGly (K)*|[8]*GlyGly (K)*[10]*GlyGly (K)*			*nd*			+
**20**	FA10_HUMAN (Coagulation factor X)	VTAFLKWIDR [6] *Acetyl (K)*		+			+	
**21**	F13A_HUMAN (Coagulation factor XIII A chain)	KETFDVTLEPLSFK [7] *Phospho (ST*)|[12] *Phospho (ST)*	*nd*			+		
		SETSRTAFGGR [6] *Phospho* (ST)	+			+		
		INETRDVLAK [10] *Acetyl* (K)		*nd*			+	
		YPQENKGTYIPVPIVSELQSGK [6] *Acetyl* (K)		+			+	
		YPQENKGTYIPVPIVSELQSGK [22] *Acetyl* (K)		+			+	
		VEYVIGRYPQENK [13] *GlyGly (K)*			+			+
**22**	F13B_HUMAN (Coagulation factor XIII B chain)	WTEPPKCIEGQEK [13] *Acetyl (K)*		+			*nd*	
**23**	HPT_HUMAN (Haptoglobin)	LPECEAVCGKPK [12] *Acetyl (K)*		*nd*			+	
		ILGGHLDAK [9] *GlyGly (K)*			+			*nd*
**24**	PLMN_HUMAN (Plasminogen)	FGMHFCGGTLISPEWVLTAAHCLEK [12] Phospho (ST)	*nd*			+		
		LSSPAVITDK [8] Phospho (ST)	+			*nd*		
		CQSWSSMTPHRHQK [14] *Acetyl (K)*		*nd*			+	
**25**	FINC_HUMAN (Fibronectin)	ISCTIANRCHEGGQSYK [4]*Phospho (ST)|*[16]*Phospho (Y)*	+			*nd*		
		IGFKLGVRPSQGGEAPR [10] *Phospho (ST)*	+			*nd*		
		FLATTPNSLLVSWQPPRAR [8] *Phospho (ST)*|[12] *Phospho (ST)*	+			+		
		TTPPTTATPIR [8] *Phospho (ST)*	+			+		
**26**	FIBG_HUMAN (Fibrinogen gamma chain)	TSEVKQLIK [2] *Phospho (ST)*	+			+		
		+TSEVKQLIK [N-term] *Acetyl (N-term*)|[5] *Acetyl (K)*		+			*nd*	
**27**	FIBA_HUMAN (Fibrinogen alpha chain)	EVVTSEDGSDCPEAMDLGTLSGIGTLDGFR [9]*Phospho (ST)*	*nd*			+		
		EVVTSEDGSDCPEAMDLGTLSGIGTLDGFR [19]*Phospho(ST)*	*nd*			+		
		EVVTSEDGSDCPEAMDLGTLSGIGTLDGFR [21]*Phospho(ST)*	*nd*			+		
		QLEQVIAKDLLPSR *Phospho* (ST)						
		MADEAGSEADHEGTHSTK [7] *Phospho (ST)*	+			+		
		MADEAGSEADHEGTHSTK [14] *Phospho (ST)*	+			+		
		MADEAGSEADHEGTHSTK [16] *Phospho (ST)*	*nd*			+		
		+GDSTFESKSYK *[N-term] Acetyl (N-term)*		+			*nd*	
		+LVTSKGDK *[N-term] Acetyl (N-term)|*[5] *Acetyl (K)*		+			*nd*	
		+VPPEWK *[N-term] Acetyl (N-term)|*[6] *Acetyl (K)*		+			*nd*	
**28**	FIBB_HUMAN (Fibrinogen beta chain)	MVSWSFHKLK [5] *Phospho (ST)*	+			*nd*		
**29**	HEMO_HUMAN (Hemopexin)	WDRELISER [7] *Phospho (ST)*	+			*nd*		
**30**	THBG_HUMAN (Thyroxine-binding globulin)	FSISATYDLGATLLK [7] *Phospho (Y)*	+			*nd*		
		FSISATYDLGATLLK [15] *Acetyl (K)*		*nd*			+	
**31**	CFAB_HUMAN Complement factor B	KEAGIPEFYDYDVALIK [17] *Acetyl (K)*		+			*nd*	
**32**	PROC_HUMAN (Vitamin K-dependent protein C)	FPCGRPWKRMEK [8] *GlyGly (K)* [12] *GlyGly (K)*			+			*nd*
		WEKWELDLDIKEVFVHPNYSK [21] *GlyGly (K)*			+			*nd*
		WEKWELDLDIKEVFVHPNYSK [3] *GlyGly (K)*			*nd*			+
**33**	RET4_HUMAN (Retinol-binding protein 4)	FSISATYDLGATLLK [7] *Phospho (Y)*	+			*nd*		
		FSISATYDLGATLLK [15] *Acetyl (K)*		+			*nd*	
		YWGVASFLQK [10] *GlyGly (K)*			+			*nd*
		FKMKYWGVASFLQK [4] *GlyGly (K)*			*nd*			+
		FKMKYWGVASFLQK [4] *GlyGly (K)*|[14] *GlyGly (K)*			*nd*			+
**34**	TRFE_HUMAN (Serotransferrin)	+SVIPSDGPSVACVKK *[N-term] Acetyl (N-term)* [14] *Acetyl (K)*		+			*nd*	
		KPVDEYK [7] *GlyGly (K)*			+			+
		KSASDLTWDNLK [12] *GlyGly (K)*			+			*nd*
**35**	TFR1_HUMAN (Transferrin receptor protein 1)	AVLGTSNFK [6] Phospho (ST)	*Protein nd*	*Protein nd*		+	*Protein nd*	
		QVDGDNSHVEMK [7] Phospho (ST)				+		
		FVMKKLNDR [4] *GlyGly (K)*|[5] *GlyGly (K)*			*Protein* ***nd***			+
**36**	VWF_HUMAN (von Willebrand factor)	LPGLHNSLVK [10] *Acetyl (K)*		+			*nd*	
		+LVCPADNLRAEGLECTK *[N-term] Acetyl (N-term)*		+			*nd*	
		+TEPMQVALHCTNGSVVYHEVLNAMECK *[N-term] Acetyl (N-term)|*[27] *Acetyl (K)*		+			*nd*	
		GLWEQCQLLK [10] *GlyGly (K)*			+			*nd*
		SLSCRPPMVK [10] *GlyGly (K)*			+			+
**37**	C1QB_HUMAN	Modified peptides	*nd*	*nd*	*nd*	*nd*	*nd*	*nd*
**Total**			*33*	*38*	*29*	*32*	*21*	*22*

Notes:

^1^ –Mascot searching with taking into account possible protein phosphorylation;

^2^ –Mascot searching with taking into account possible protein acetylation;

^3^ –Mascot searching with taking into account possible protein ubiquitination; *nd*—not detectable;

*—max missed cleavages: 2

In contrast, the number of phosphopeptides did not change in GBM compared to control samples. Most phosphopeptides identified in control and GBM samples were quantified. Only the peptide KEFMSQGNK attributed to the complement C1r subcomponent appeared only in 11 samples (three control and eight GBM) under Phospho-sensitive search conditions (S10 Table). Despite of it, Progenesis LC-MS software recruited this phosphopeptide for quantitative analysis of complement C1r in both types of plasma obtained from control and diseased patients.

For the complement C1q subunit B (C1QB) there were no modified peptides at all. For some proteins, modified peptides that encompassed PTMs of one type (Table 3) were identified. For example, FIBB, HEMO, FINC and APOA were associated exclusively with phosphopeptides. Eight proteins, CRP, APOA1, CFAB, C1S, CERU, F13B, THBG and FA10, contained acetylated peptides only. CHLE, APOB, FA9, C1R, RET4 and F13A, carried PTMs of all three categories. The most abundant case was simultaneous observation of two different PTM types in the same protein (but different peptides), for example, acetylation and ubiquitination or phosphorylation and acetylation. Such generalization was true for high-abundant albumin, as well as low-abundant von Willebrand factor (VWF_HUMAN).

C1R, THBG, FIBG and RET4, contained peptides with more than a single modification, that is, a single peptide incorporated two modified sites (e.g. phosphorylated and ubiquitinated at the same time) (Table 3). For example, peptide KEFM*S*QGN*K* (C1R) comprised ubiquitination at site [9]*K* together with phosphosite [5]*S*, which were detected in eight of 12 GBM samples. Another four samples contained peptide KEFMSQGNK with only ubiquitination at site [9]*K*. In addition, there was a peptide FLDSNIK from apolipoprotein B-100, where two different modifications occurred at the same amino acid residue. This observation is consistent with Chick et al. [8], who observed, mono-, di- and tri-methylation jointly with acetylation of the same site. Choudhary et al. [30] has reported that every peptide present after digestion exhibits 8–12 modified forms, supposing multiplex modifications at the same site. For such multiplex-modified peptides, we observed peculiarities in quantitative analysis. For example, the peptide FLDSNIK with ubiquitination at site [7]*K* and acetylation at the same site [7]*K* was detected in 12 GBM samples but expressed quantitative differences between individuals. The peptide average normalized abundance in GBM samples nos. 13–16 and 22 was ˃10-fold compared to that in samples nos. 14–21 and 23, and 73-times higher than in sample no. 24.

**Fig 3:**
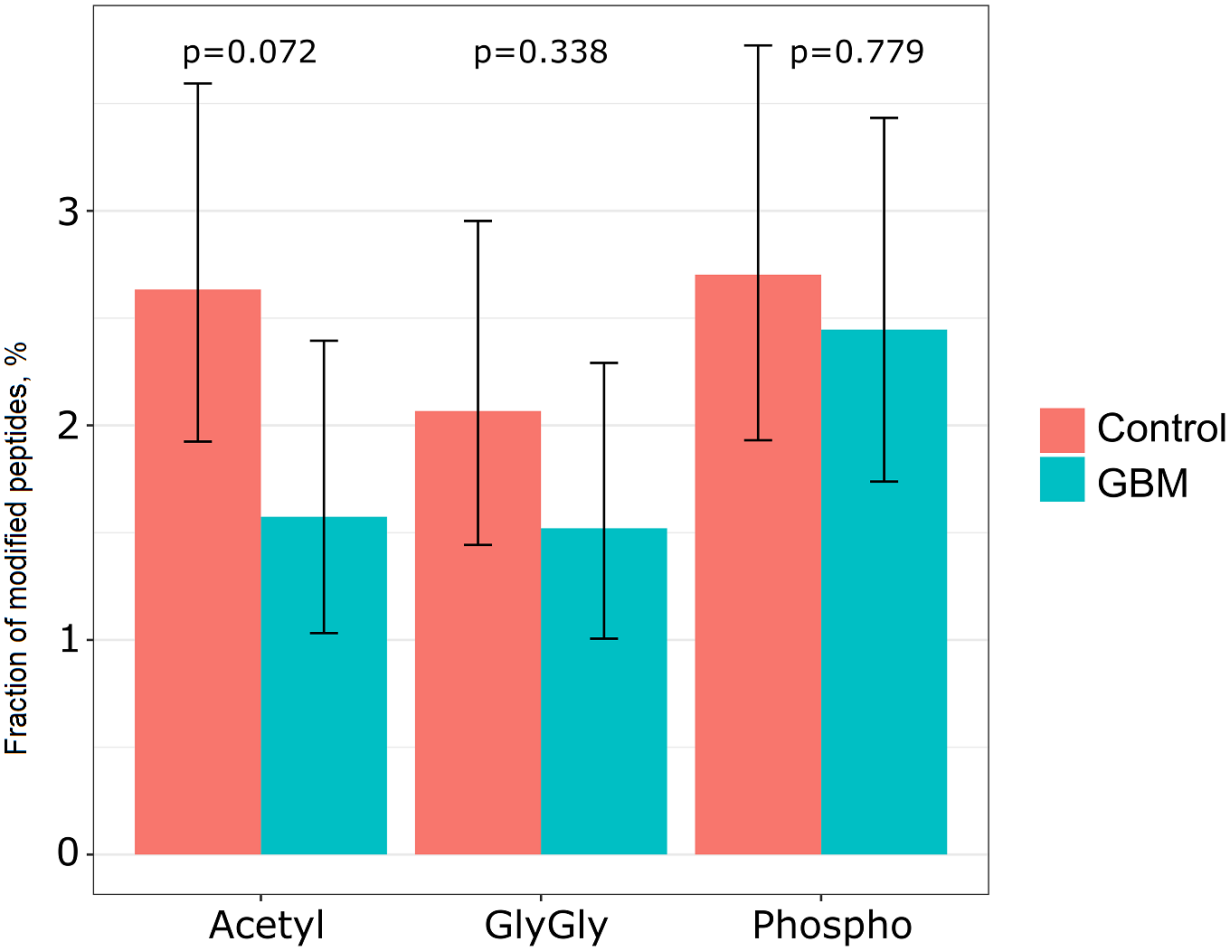
Changes in the fractions of PTM peptides of FDA-approved plasma biomarkers in GBM compared to control.

Changes in the occurrences of PTM peptides of FDA-approved plasma biomarkers are present in Fig 3. The difference between the portions of Phospho, Acetyl and GlyGly peptides (bar height is proportional to the fraction of modified peptides) for GBM versus control was not significant (p = 0.779, 0.072 and 0.338, respectively). However, analysis of acetylated peptides demonstrated that 15 (40%) of 38 acetylated peptides (Table 3) carried N-terminal (Nt) acetylation. These peptides matched eight FDA-approved biomarkers (ALBU, APOA1, APOB, C1R, FIBA, FIBG, TRFE, and von Willebrand factor). Progenesis LC-MS quantified all of these Nt-acetylated peptides in all 12 control plasma samples. We observed a reduction of 100% in Nt-acetylated peptides in 12 GBM plasma samples (S11 Table).

Some studies have addressed Nt-acetylation and ubiquitination in high-grade glioma [31, 32]. Nt acetylation is one of the most common covalent modifications in eukaryotes, and dysregulation of this PTM is a trait of many human cancers [31, 33]. The data presented in the paper are in accordance to the known literature on a central part of the pathway in various malignancies, including glioblastoma. A lot of evidence indicates that Nα-terminal acetyltransferases, which are dysregulated in numerous human cancers, can serve as therapeutic targets [33]. Thus, a reduced number of Nt-acetylated peptides may have important therapeutic implications for GBM.

### PTM-sensitive patterns, specific for the FDA-approved plasma biomarkers

We selected PTM patterns that distinguished GBM datasets from the control ones. The GBM-negative pattern was associated with the presence of a modified peptide in the control plasma and a corresponding absence of the same peptide in GBM samples. Conversely, the GBM-positive status denoted the absence of a modified peptide in control plasma and its presence in GBM.

To illustrate the distribution of PTM patterns, we mapped our results onto the list of 37 FDA-approved plasma proteins identified in the control and GBM samples (Table 4). Sixty-five modified peptides resembled the GBM-negative pattern, while 40 others exhibited the PTM-positive pattern. About half of the proteins (17) matched both GBM-positive and GBM-negative patterns; 15 proteins resembled exclusively GBM-negative patterns; and three proteins, namely transferrin receptor protein 1, complement C1s and coagulation factor IX, were GBM-positive. Coagulation factor X (FA10_HUMAN) did not contain PTM peptides that could be used for separating control against GBM.

**Table 4:** GBM-negative and GBM-positive patterns’ counted for FDA-approved plasma proteins.

## No.	Protein name	Mass, kDa	No. of PTM patterns							
			Phospho (S, T, Y)		Acetyl (K, protein N-term)		GlyGly (K)*		Total	
			GBM-negative	GBM-positive	GBM-negative	GBM-positive	GBM-negative	GBM-positive	GBM-negative	GBM-positive
**1**	Apolipoprotein B-100	518	4	4	4	2	4	2	12	8
**2**	Apolipoprotein(a)	501	1	2	*nd*	*nd*	*nd*	*nd*	1	2
**3**	von Willebrand factor	309	*nd*	*nd*	3	*nd*	1	*nd*	3	*nd*
**4**	Fibronectin	269	2	*nd*	*nd*	*nd*	*nd*	*nd*	2	*nd*
**5**	Complement C4-A	196	2	*nd*	1	1	*nd*	*nd*	3	1
**6**	Complement C5	188	*nd*	*nd*	*nd*	*nd*	4	*nd*	4	*nd*
**7**	Complement C3	187	2	*nd*	2	*nd*	*nd*	*nd*	4	*nd*
**8**	Alpha-2-macroglobulin	163	*nd*	*nd*	1	1	*nd*	*1*	1	2
**9**	Ceruloplasmin	122	*nd*	*nd*	1	*nd*	*nd*	*nd*	1	*nd*
**10**	Fibrinogen alpha chain	96	*nd*	4	3	*nd*	*nd*	*nd*	3	4
**11**	Plasminogen	91	*1*	1	*nd*	*1*	*nd*	*nd*	2	1
**12**	Transferrin receptor protein 1	88	*nd*	2	*nd*	*nd*	*nd*	*1*	*nd*	3
**13**	Complement factor B	86	*nd*	*nd*	*1*	*nd*	*nd*	*nd*	1	*nd*
**14**	Coagulation factor XIII A chain	84	*nd*	1	*1*	*nd*	*nd*	*nd*	1	1
**15**	Complement C1r subcomponent	83	*nd*	*nd*	1	*nd*	*nd*	2	1	2
**16**	Complement C1s subcomponent	77	*nd*	*nd*	*nd*	*1*	*nd*	*nd*	*nd*	1
**17**	Serotransferrin	77	*nd*	*nd*	1	*nd*	*1*	*nd*	2	*nd*
**18**	Coagulation factor XIII B chain	76	*nd*	*nd*	1	*nd*	*nd*	*nd*	1	*nd*
**19**	Serum albumin	73	*nd*	*nd*	2	1	1	*nd*	3	1
**20**	Cholinesterase	68	*nd*	*nd*	*nd*	*nd*	1	*nd*	1	*nd*
**21**	Biotinidase	62	*1*	*1*	*nd*	*nd*	*nd*	*1*	1	2
**22**	Fibrinogen beta chain	57	1	*nd*	*nd*	*nd*	*nd*	*nd*	1	*nd*
**23**	Alpha-2-antiplasmin	55	*nd*	*nd*	*nd*	*nd*	*1*	*nd*	1	*nd*
**24**	Coagulation factor X	55	*nd*	*nd*	*nd*	*nd*	*nd*	*nd*	*nd*	*nd*
**25**	Antithrombin-III	53	*nd*	*nd*	*1*	*1*	*nd*	*nd*	1	1
**26**	Coagulation factor IX	52	*nd*	*1*	*nd*	*1*	*nd*	*nd*	*nd*	2
**27**	Hemopexin	52	*1*	*nd*	*nd*	*nd*	*nd*	*nd*	1	*nd*
**28**	Vitamin K-dependent protein C	52	*nd*	*nd*	*nd*	*nd*	*2*	*1*	2	1
**29**	Fibrinogen gamma chain	51	*nd*	*nd*	*1*	*nd*	*nd*	*nd*	1	*nd*
**30**	Thyroxine-binding globulin	47	*nd*	*1*	*1*	*nd*	*1*	*1*	2	2
**31**	Alpha-1-antitrypsin	47	*nd*	1	1	*nd*	1	1	2	2
**32**	Haptoglobin	45	*nd*	*nd*	*nd*	*1*	1	*nd*	1	1
**33**	Apolipoprotein A-I	31	*nd*	*nd*	1	*nd*	*nd*	*nd*	1	*nd*
**34**	Complement C1q subcomponent subunit C	26	*nd*	*nd*	*nd*	*nd*	*1*	1	1	1
**35**	Complement C1q subcomponent subunit B**	27	*nd*	*nd*	*nd*	*nd*	*nd*	*nd*	*nd*	*nd*
**36**	C-reactive protein	25	*nd*	*nd*	*1*	*nd*	*nd*	*nd*	1	*nd*
**37**	Retinol-binding protein 4	23	*1*	*nd*	*1*	*nd*	*1*	*2*	3	2

* maximum missed cleavages: 2.

** does not contain modified peptides.

The number of patterns did not depend on the number of the high-scoring peptides identified by the MS/MS method for each protein. Fibronectin was identified by ˃65 high-quality peptides that delivered only two Phospho-negative patterns, while RET4 identified by 10 peptides had three GBM-negative and two GBM-positive patterns (Table 4). Fibrinogen exhibited no other pattern except for the GBM-negative phosphorylation. Serum albumin did not affect the patterns linked to phosphorylation, while acetylation was present in three patterns, and one pattern was attributed to ubiquitination.

As shown in Table 4, there was no a relationship between the molecular masses of the proteins and the number of PTM-sensitive patterns in them, that is, the proteins with comparable masses had different PTM patterns. For example, apolipoprotein B-100, the abundant plasma species weighing ˃0.5 MDa, collected 20 patterns, whereas apolipoprotein(a) with comparable mass had only three. Also, C-reactive protein and retinol-binding protein 4, which are of similar weight (25 and 23 kDa, respectively), were quite different single modified peptide detected in C-reactive protein versus five pattern-making peptides revealed for the retinol-binding protein (see Table 4).

We identified disequilibrium of Nt-acetylated and ubiquitinated proteins in GBM, as compared to the control samples. This suggests that aberrant de-modifications of plasma proteins could happen in glioblastoma. At the proteome level, it was observed that, in disease, it is easier to lose than gain, at least in regard to PTMs.

## Conclusions

The simple method to perform PTM-mining using embedded options of MS/MS search engine was investigated using GBM as an illustrative example. Application of a Mascot search for single variable protein/peptide PTMs, such as phosphorylation, acetylation or ubiquitination, increased the fraction of the proteins identified by ≥ 3 peptides for GBM spectra. A database search gave an opportunity to enrich the sequence coverage for the given plasma protein by gathering peptides from several search runs with variable modifications. The higher-level modification of proteins in the GBM plasma was observed in comparison with the control samples. In the majority of cases, the distinction of GBM patterns was due to a sufficiently lower number of acetylated and ubiquitinated peptides in GBM. We found a reasonable option to unravel modified peptides that exhibit disease-specific patterns of PTMs. The most notable changes were found in relation to Nt-acetylated peptides. It is known that alterations of proteins Nt-acetylation correlate to a low survival rate and aggressiveness of various tumors [33]. We suggest that decrease or increase in the number of modified plasma proteins (PTM-sensitive patterns) could serve as a disease-unspecific indicator of a health threat. It was demonstrated by using up-to-date MS/MS that such changes could be easily captured at the level of the high-abundant FDA-approved proteins markers, in contrast with the current concept of biomarker discovery by MS with high-sensitivity targeted methods applied to a small number of candidate markers. Notwithstanding, the obtained data are correlated with the results of Geyer et al [34], who suggested a strategy of plasma proteome profiling consisting of the measurement of large numbers of plasma proteins at the greatest possible depth. These high-dimensional profiles could indicate current disease risk as well as efficacy of lifestyle changes or pharmacological interventions.

## Supporting information

S1 Table List of proteins identified by ≥ 3 high quality peptidesby Mascot search without variable post-translational modifications in control. (XLSX)

S2 Table List of proteins identified by ≥ 3 high quality peptides by Mascot search with possible protein phosphorylation in control. (XLSX)

S3 Table List of proteins identified by ≥ 3 high quality peptides by Mascot search with possible protein acetylation in control. (XLSX)

S4 Table List of proteins identified by ≥ 3 high quality peptides by Mascot search with possible protein ubiquitination in control. (XLSX)

S5 Table List of proteins identified by ≥ 3 high quality peptides by Mascot search without variable post-translational modifications in GBM. (XLSX)

S6 Table List of proteins identified by ≥ 3 high quality peptidesby Mascot search with possible protein phosphorylation in GBM. (XLSX)

S7 Table List of proteins identified by ≥ 3 high quality peptides by Mascot search with possible protein acetylation in GBM. (XLSX)

S8 Table List of proteins identified by ≥ 3 high quality peptides by Mascot search with possible protein ubiquitination at GBM. (XLSX)

S9 Table Number of the identified proteins in human plasma of healthy individuals (Control) and glioblastoma patients (GBM) depending on the possible post-translational modifications (PTMs). (DOC)

S10 Table Phosphorylated peptides of FDA-approved biomarkers in control and GBM plasma samples. (XLSX)

S11 Table Nt-acetylated peptides of FDA-approved biomarkers in control and GBM plasma samples. (XLSX)
