# Supplementary material for: Post-translational modifications of FDA-approved plasma biomarkers in glioblastoma samples
Source: PLoS One. 2017 May 11;12(5):e0177427. doi: 10.1371/journal.pone.0177427 (PMC5426747; doi:10.1371/journal.pone.0177427)
Supplement: S9 Table — (DOC) [file pone.0177427.s009.doc]

S9 Table. Number of the identified proteins in human plasma of healthy individuals (Control) and glioblastoma patients (GBM) depending on the possible post-translational modifications (PTMs)

| Column/Parameter | Control | | | | GBM | | | |
| --- | --- | --- | --- | --- | --- | --- | --- | --- |
|  | #1  no PTMs^1^ | #2  Phospho^2^ (S,T,Y) | #3 Acetyl (K, protein N-term)^3^ | #4  GlyGly(K)^4,*^ | #5  no PTMs^1^ | #6  Phospho^2^ (S,T,Y) | #7 Acetyl (K, protein N-term)^3^ | #8  GlyGly(K)^4,*^ |
| FDR^**^ | 0.80 % | 0.74 % | 0.82 % | 0.87 % | 0.57 % | 0.62 % | 0.72 % | 0.82 % |
| Total number of peptide identifications | 3227 | 3649 | 5871 | 5002 | 4642 | 3418 | 3742 | 4642 |
| Number of quantified peptides | 2222(69%) | 2241(61%) | 2122(36%) | 2208(44%) | 2458(53%) | 2357(69%) | 2363(63%) | 2458(53%) |
| Total number of protein identifications | 446 | 519 | 878 | 739 | 597 | 402 | 466 | 597 |
| Number of proteins identified by 1 peptide | 141 (32 %) | 157 (30%) | 240 (27%) | 219 (29%) | 170 (28 %) | 109 (27 %) | 126 (27%) | 171 (29%) |
| Number of proteins identified by 2 peptides | 38 (8 %) | 44 (8.5 %) | 72 (8%) | 65 (9%) | 53 (9 %) | 49 (12 %) | 55 (12%) | 50 (8%) |
| Number of unquantified proteins | 142 (32 %) | 181 (35%) | 428 (49%) | 317 (43%) | 236 (39 %) | 117 (29 %) | 155 (33%) | 238 (40%) |
| Number of proteins identified by ≥ 3 peptides | 125 (28 %) | 137 (26.5%) | 138 (16%) | 138 (19%) | 138 (23 %) | 127 (32 %) | 130 (28%) | 138 (23%) |
| Mascot score  Mean ± SD | 1245 ± 1865  (20 ̶ 14600) | 1260 ± 1987  (16 ̶ 15800) | 1328 ± 2135  (22 ̶ 16800) | 1320 ± 2088  (22 ̶ 16400) | 1554 ± 2531  (17 ̶ 19400) | 1490 ± 2276  (15 ̶ 17200) | 1570 ± 2457  (17 ̶ 18900) | 1432 ± 2027  (17 ̶ 19400) |
| Sequence coverage, %^***^, Mean ± SD | 37 ± 22  (1 ̶ 91) | 35 ± 23  (1 ̶ 91) | 34 ± 23  (1 ̶ 91) | 36 ± 23  (1 ̶ 91) | 34 ± 25  (1 ̶ 95) | 37 ± 24  (1 ̶ 95) | 36 ± 24  (1 ̶ 95) | 35 ± 25  (1 ̶ 95) |
| Median | 35 | 33 | 31 | 33 | 32 | 34 | 34 | 32 |

Notes: 1 – Mascot searching without definition of possible protein post-translational modifications (PTMs); 2 – Mascot searching with taking into account possible protein phosphorylation; 3 – Mascot searching with taking into account possible protein acetylation; 4 – Mascot searching with taking into account possible protein ubiquitination

* ̶ max missed cleavages: 2; ** ̶ FDR (false discovery rate) was calculated by Mascot percolator;

*** ̶ seq.cov. for proteins identified by ≥ 3 peptides, 14 аа (6 ̶ 31 аа) the length of one peptide, on average
